# Environmental Influences on Kelp Performance across the Reproductive Period: An Ecological Trade-Off between Gametophyte Survival and Growth?

**DOI:** 10.1371/journal.pone.0065310

**Published:** 2013-06-03

**Authors:** Margaret B. Mohring, Gary A. Kendrick, Thomas Wernberg, Michael J. Rule, Mathew A. Vanderklift

**Affiliations:** 1 School of Plant Biology and UWA Oceans Institute (M096), The University of Western Australia, Crawley, Western Australia, Australia; 2 Australian Institute of Marine Science, Crawley, Western Australia, Australia; 3 Department of Environment and Conservation, Kensington, Western Australia, Australia; 4 CSIRO Wealth from Oceans Flagship, Floreat, Western Australia, Australia; University of New South Wales, Australia

## Abstract

Most kelps (order Laminariales) exhibit distinct temporal patterns in zoospore production, gametogenesis and gametophyte reproduction. Natural fluctuations in ambient environmental conditions influence the intrinsic characteristics of gametes, which define their ability to tolerate varied conditions. The aim of this work was to document seasonal patterns in reproduction and gametophyte growth and survival of *Ecklonia radiata* (C. Agardh) J. Agardh in south-western Australia. These results were related to patterns in local environmental conditions in an attempt to ascertain which factors explain variation throughout the season. *E. radiata* was fertile (produced zoospores) for three and a half months over summer and autumn. Every two weeks during this time, gametophytes were grown in a range of temperatures (16–22°C) in the laboratory. Zoospore densities were highly variable among sample periods; however, zoospores released early in the season produced gametophytes which had greater rates of growth and survival, and these rates declined towards the end of the reproductive season. Growth rates of gametophytes were positively related to day length, with the fastest growing recruits released when the days were longest. Gametophytes consistently survived best in the lowest temperature (16°C), yet exhibited optimum growth in higher culture temperatures (20–22°C). These results suggest that *E. radiata* releases gametes when conditions are favourable for growth, and *E. radiata* gametophytes are tolerant of the range of temperatures observed at this location. *E. radiata* releases the healthiest gametophytes when day length and temperature conditions are optimal for better germination, growth, and sporophyte production, perhaps as a mechanism to help compete against other species for space and other resources.

## Introduction

Seaweeds of the order Laminariales (kelps) are the major habitat-forming species on most temperate subtidal reefs [1], [2]. Kelps provide habitat and food [3]–[5] to a wide range of organisms, including a large number of commercially important species, such as fish and lobsters [6]. Around the globe, kelps are at risk of decline due to anthropogenic and environmental stressors [7]–[9], and so these ecologically significant species' have become a matter of interest [10]; particularly with respect to their diplo-haplontic life cycle characterised by filamentous microscopic gametophytes and macroscopic sporophytic lamina. Compared to the intensely studied macroscopic sporophytes, substantially fewer studies have examined the microscopic gametophyte and early sporophyte stages, and these parts of the kelp life cycle have been identified as a bottleneck in the current understanding of the ecology of kelp populations [6], [11]. With existent threats to kelp populations becoming more apparent, knowledge of the natural variability in reproduction and juvenile performance is necessary to determine how changing pressure will affect populations of these habitat-forming organisms.

Laminariales have a heteromorphic lifecycle which alternates between a microscopic, gametophyte phase (N), and a macroscopic sporophyte phase (2N) [12]. Adult kelps, or sporophytes, produce zoospores in specialised tissue called sporophylls, or sori, and the timing of production, and subsequent release of zoospores, is highly variable between genera, species, and populations [13], [14]. Some genera, such as *Macrocystis*, reproduce year-round [15], [16], while many other species reproduce in distinct seasons [6], [17]. Zoospore production for *Macrocystis* spp. occurs when nutrient levels are optimum, and coincides with ideal temperature and light conditions [15], [18]. In other Laminariales, the subsequent release of zoospores into the environment has been linked to environmental cues such as changes in water temperature and light [17], [19], or storm events [20] and the resultant abrasion and erosion of thallus tissue [21]. Once zoospores are released from the sori they actively swim until they find a suitable substrate to settle onto and begin to grow as a gametophyte [22]. Gametophytes grow for up to three weeks before fertilisation is possible, and the sporophyte grows from the fertilised egg still surrounded by the female gametophyte [23].

Many studies have demonstrated that, under laboratory conditions, kelp gametophytes are highly sensitive to changes in water temperature [24], [25]. For example, the gametophytes of three species of *Laminaria* from the North Sea were able to survive in 20°C water for one week, but could not tolerate a 1°C increase over the same time period [26]. At the extremes of their temperature tolerance, gametophytes exhibit reduced growth, limited fertilisation success and die without producing sporophytes [26], [27]. Patterns in temperature tolerance are often a product of a species' ability to acclimate to ambient environmental conditions and may vary across the distribution of kelp [27], [28]. However, very little is known about whether temperature changes affect gametophyte performance consistently across the reproductive period.

The intrinsic characteristics of zoospores, gametophytes and early life stages of kelp can vary temporally [29]. Tala *et al.* [29] found that while *Lessonia trabeculata* was fertile year-round, gametophytes would only produce gametes, and sporophytes would only recruit successfully, when zoospores were released during autumn. Seasonal variation in juvenile performance was assumed to be dependent on the intrinsic characteristics of the zoospores [29]. Tala *et al.* [29] speculated that zoospores and gametophytes obtained in different seasons of the year may have different light and temperature requirements for growth and development [29], [30] and suggested that their study fell short of fully evaluating possible seasonal patterns, because this required special manipulation of the culture conditions to simulate environmental variation [29]. Despite the broad understanding of these seasonal patterns, very little is known about temporal variation in gametophyte growth and survival over smaller temporal scales, such as weeks and months across the reproductive period of a seasonally limited species.

The most extensive habitat-forming macroalga in temperate Australasia is the kelp *Ecklonia radiata*, and it plays a critical role in most cool water ecosystems and food webs [5], [31], [32]. Australian kelp forests support fish and invertebrate communities [33]–[37], as well as algal communities [3], [38], [39]. Despite the ecological significance of kelp to marine communities in southern Australia, the reproductive biology of *E. radiata* in Australia is poorly understood [40], [41]. Novaczek [27] found that the reproduction of gametophytes from New Zealand was retarded outside the optimum temperatures of 9 to 24°C, and that mortality occurred below 8°C and above 26°C. Novaczek [27] also found temporal variation in the performance of gametophytes over the year, with gametophytes collected in summer able to produce ova within 16 days of release, while in winter it took more than 25 days [27]. Given that the distribution of *E. radiata* is between 27°43′S–35°11′S in Western Australia, within a sea temperature range of 11–26°C [42], this species exists close to, and sometimes beyond, these extremes, suggesting that the period of reproduction should occur at distinct periods in the year when ambient temperatures are within tolerable levels.

Given the dearth of knowledge regarding the small scale variability in gametophyte production and performance, we aimed to examine *E. radiata* zoospore production, settlement densities, gametophyte growth and survival across the observed seasonal reproductive period (January–April). We hypothesised that there would be a level of temporal variation in gametophyte performance over the reproductive season and that this would be related to changes in *in situ* environmental conditions (sea temperature, day length, light intensity, wave action and lunar cycle). We also hypothesized that there would be an interactive effect of collection time and water temperature on gametophyte growth and survival.

## Methods

In order to test the hypothesis that *Ecklonia radiata* gametophytes in Western Australia exhibit temporal variation in early performance it was necessary to measure zoospore production, settlement densities, and gametophyte growth and survival across the period of reproduction. Kelps for zoospore release were collected every second week over a period of five months, encompassing the time when *E. radiata* is known to be fertile [43]. During this time, zoospores were available for experiments for 3.5 months (sampled every two weeks for a total of seven sampling times) from mid-austral summer (January) to the end of autumn (April).

**Figure 1:**
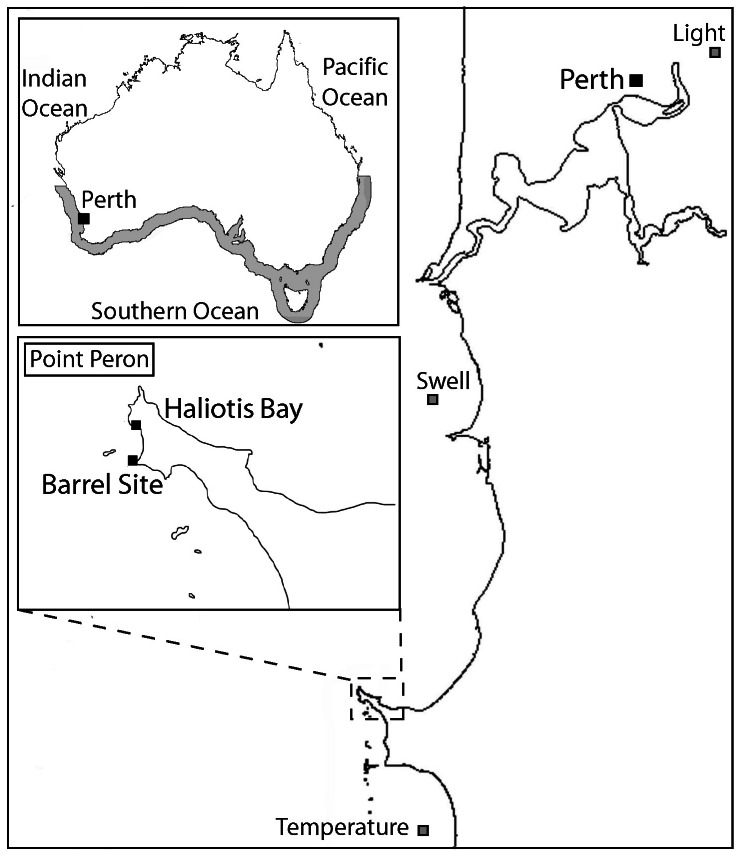
Collection sites for two-weekly zoospore counts and growth experiment. Main map - Perth region, Western Australia; Upper Inset – Australia map showing oceans and distribution of *Ecklonia radiata* in grey; Lower Inset - Point Peron, displaying Barrel Site and Haliotis Bay collection sites.

Two collection sites (Barrel Site and Haliotis Bay North), separated by approximately 500 m, were sampled at Point Peron, within the Shoalwater Islands Marine Park, Western Australia (Figure 1). To sample within these sites, all necessary Western Australian Department and Environment and Conservation, and Department of Fisheries permits were obtained prior to field work. Although relatively close, the sites were considerably different with respect to environmental characteristics. Barrel Site has a north facing aspect, and is situated along the edge of a channel where water flow is constant. Haliotis Bay North has a southerly aspect, is relatively protected and calm, and has extensive sand inundation. Both collection areas have dense kelp beds growing on limestone reef, between 2–4 m depth. Eight *E. radiata* thalli were haphazardly selected and harvested from each site. Thalli were collected by a diver by cutting the stipe just above the holdfast. Thalli were stored in a damp, labelled calico bag, which was wrapped in plastic and kept on ice for transport to the laboratory (<6 hrs).

In the laboratory, four thalli were selected from each site to be used for zoospore quantification. The laterals were cut away from the central lamina, which was blotted dry with paper towels. Ten 27 mm diameter discs of tissue (total surface area 11451.1 mm^2^) were punched from each central lamina with a PVC corer, starting at the distal end of the thallus. The discs were then allowed to desiccate at room temperature for 1 hr. The discs from each lamina were then placed in a cup (one for each thalli) containing 50 mL of seawater and gently stirred over a 20 min period to encourage zoospore release. After 20 minutes, 10 mL of zoospore solution was added to a vial with 0.1 mL of 70% ethanol (to sedate the zoospores) and vigorously shaken to ensure adequate mixing. Three 1 mL samples from each vial of zoospore solution were dropped onto Neubauer counting chambers, and the number of zoospores in two, 1 mm (0.1 µL) grids were counted, resulting in a total of six grids per replicate *E. radiata* thallus. The results of the six counts were averaged for analysis.

The remaining thalli from both sites, as well as the left over tissue and laterals from the thalli used for zoospore counts, were combined as a zoospore source for the gametophyte culture experiment. A total of 60–100 g of reproductive tissue was collected from the combined plants. With careful examination this tissue could be identified as slightly raised, discoloured patches. For each site a large plastic container was lined with 60 labelled slides, and filled with 5 L of freshly collected seawater. After 1 hr of desiccation all reproductive tissue collected was submerged in the container and carefully agitated over the following hour. The slides and reproductive tissue were then left undisturbed for 18 hrs so that zoospores could settle on, and adhere to, the slides.

Twenty small aquaria were filled with 5 L of Provasoli-Enriched seawater and randomly allocated to two controlled environment rooms (set at 16°C, light over both rooms between 10–15 µE m^−2^ s^−1^; photoperiod 12 h light∶12 h dark). Provasoli solution contains increased levels of nitrate and phosphate, a TRIS base buffer, trace metals and vitamins meaning that the seawater for algae culture is not nutrient limiting [44] and in this case was added to ensure any temporal changes in growth and survival would not be confounded by seasonal shifts in water quality. Small heaters were used to heat the aquaria to 16, 18, 20, and 22°C, with five replicates per temperature. These treatments were selected to encompass all temperatures that *E. radiata* are subjected to throughout its distribution in Australia [42]. After the 18 hr settlement period, two slides were randomly selected and allocated to each aquarium. The remaining slides were used as the initial (Day 1) sample prior to treatment. One slide was randomly selected from each aquarium after three and six days, respectively. Slides were examined under a compound microscope and six random photographs (0.17×0.13 mm) were taken from each slide. Gametophyte densities were collected from each photo, and the area of one individual per photo (30 per treatment) was measured using ImageJ software [45]. Effects on survival and growth were inferred from differences in relative density and size after six days

**Figure 2:**
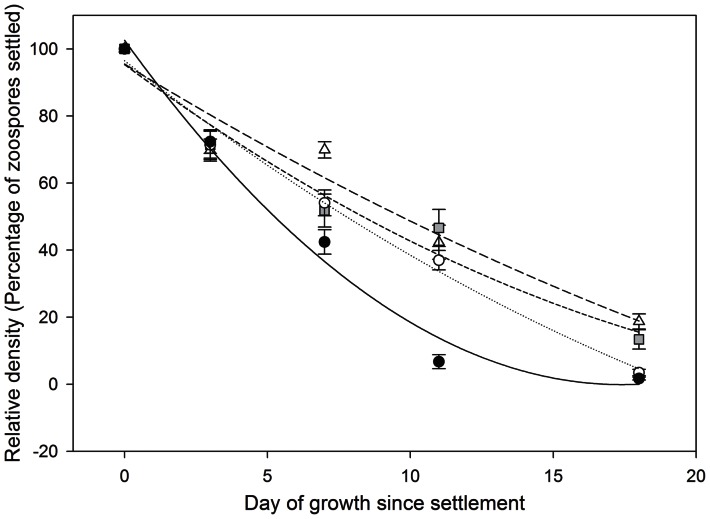
Mean survival (±SE) over 18 days from samples collected on the 24 January 2011. Gametophytes were grown in four different temperatures; 16°C (white triangle, long dash line), 18°C (grey square, small dash line), 20°C (white circle, dotted line), 22°C (black circle, solid line). Lines shown are quadratic regressions.

The first time gametophyte culturing was carried out, 100 slides were seeded with zoospores, and slides were allocated to each treatment so that there were five sample times; days 1, 3, 7, 11, and 18 (Figure 2). The purpose of this test was to determine the time in culture required to detect differences in survival between temperatures. Significant differences in density (F_(3,116)_ = 8.98, *p*<0.001) with temperature were recorded after only seven days, and so all subsequent cultures were carried out for 6 days..

Environmental data were collected over the reproductive season and compared against patterns in *E. radiata* reproduction. Data included sea temperature (°C), significant wave height (m), solar radiation (MJ m^−2^), day length (photoperiod, hrs) and lunar phase. Sea temperature data were measured daily in Warnbro Sound near Rockingham (Western Australian Department of Fisheries, unpubl. data). Wave height was measured every 30 mins at Owen Anchorage (Western Australian Department of Transport, unpubl. data). Day-length was provided by Geoscience Australia, and solar radiation was measured daily at Perth airport by the Australian Bureau of Meteorology. All data were averaged for a weekly result for best comparison to zoospore densities. The dates of the full moons for each month (Sutherland Shire Weather Station, unpubl. data) were also included in the comparisons.

Univariate analyses were used to test whether the initial zoospore density, zoospore size and percentage of germinated zoospores (hereafter termed germination success) varied among collection dates. Zoospores were judged to have successfully germinated if a germ tube had visibly extended from the zoospore within 18 hrs of release [41]. Pearson Product-Moment correlation (Pearson's r) was used to determine if there was an effect of spore release density, settlement density and spore size on the results of the culture experiment. Two-way Analysis of Variance (ANOVA) was used to determine whether there was a difference in gametophyte survival and growth among zoospore sample periods (random factor) and culture temperatures (fixed factor). Prior to analysis, homogeneity of variances was tested using Cochran's test (density day 3 *p*<0.01, day 6 *p*<0.01; after log transformation day 3 *p* = 0.110, day 6 *p* = 0.079. Growth day 3 *p* = 0.097, day 6 *p* = 0.129, no transformation required). Pairwise comparisons could not be carried out on the individual ANOVA terms as significant interactions between sample date and temperature occurred in all cases [46]; however, where a significant interaction was returned, pairwise Student Newman-Keuls comparisons were used to investigate differences between temperatures (fixed) within each sample date (random). Regressions were used to examine the strength of relationships between initial zoospore density, size, germination and gametophyte survival and growth and environmental variables. All analyses were carried out using GMAV [47] and Minitab [48].

## Results

**Figure 3:**
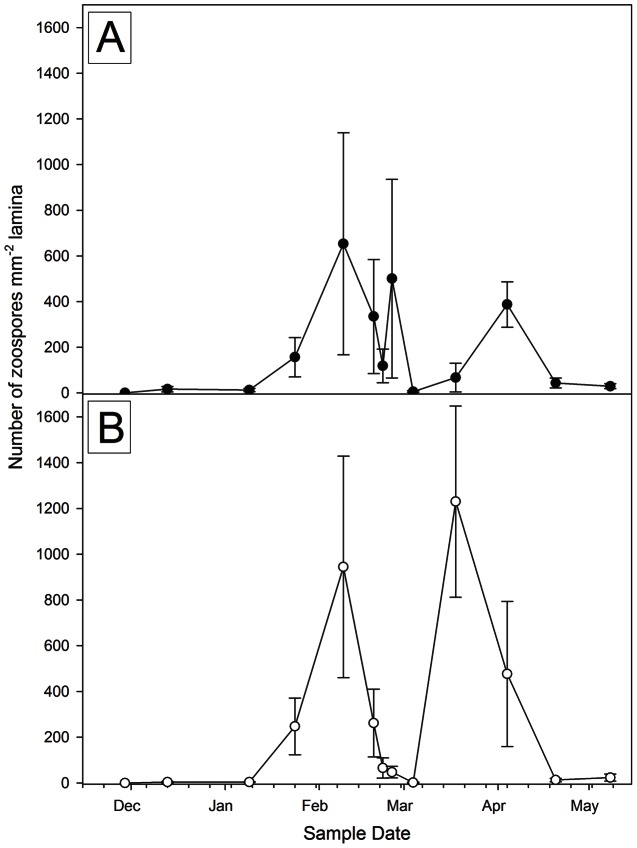
Mean (±SE) number of zoospores in *Ecklonia radiata* plants measured at two-weekly intervals for five months. Number of zoospores measured per mm of lamina tissue, five month sampling period from November 2010 to May 2011. Two sites were Barrel Site (Plot A; black, solid circles) and Haliotis Bay North (Plot B; white, hollow circles).

*Ecklonia radiata* thalli released zoospores between 24^th^ January (sample period 1) and 20^th^ April (sample period 7), 2011 (Figure 3). There were two peaks of zoospore production at Haliotis Bay, with a maximum number of zoospores recorded on March 18^th^ (sample period 5). The maximum number of zoospores recorded at Barrel Site occurred on the 19^th^ February (sample period 3).

### Initial settlement results

**Figure 4:**
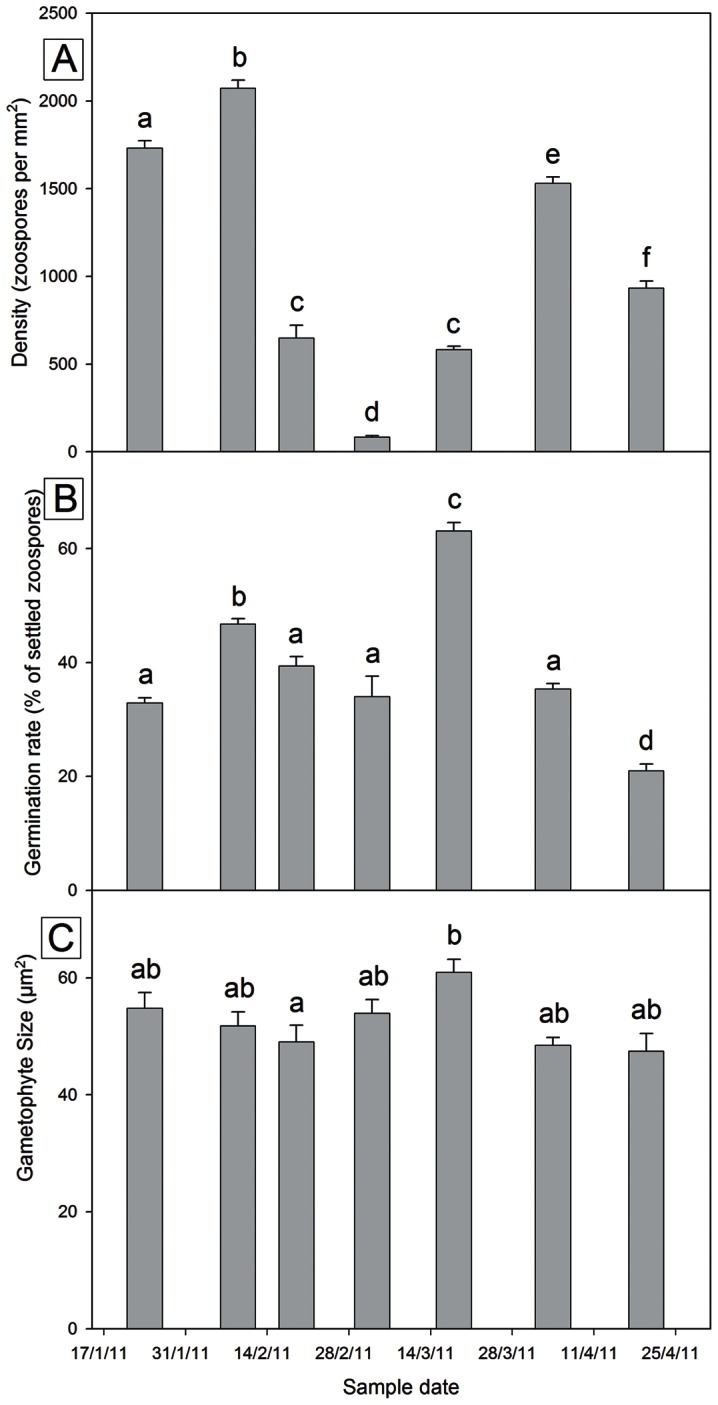
Plots of mean (±SE) measurements taken 18 hours after release. Plot A) –settlement density, B) gamete size, and C) percentage of initial density which had germinated. Within each plot, bars were allocated the same letters if they were not statistically significantly different (P>0.05).

There were significant differences in the settlement density (F_(6, 833)_ = 289.37, *p*<0.001), percentage of germinated gametophytes (F_(6, 833)_ = 3.47, *p*<0.001), and gametophyte size (F_(6,133)_ = 64.28, *p*<0.001) between sampling periods across this study (Figure 4). Initial gametophyte densities peaked twice over the sample period with densities reaching a maximum on the second (9^th^ Feb) sample period, while the minimum density was recorded mid-season. All initial densities were significantly different between sample times except those samples collected on the fourth (4^th^ Mar) and sixth (4^th^ Apr) sample periods. There was a trend between initial settlement densities (Figure 4A) and zoospore concentrations released from the lamina; however this was not a significant correlation (Pearson's r = 0.724, *p* = 0.066, n = 7). The number of zoospores that had germinated after 18 hrs (Figure 4B) did not reflect patterns in initial settlement densities as germination success was not correlated with zoospore concentration (Pearson's r = 0.046, *p* = 0.923, n = 7) or initial settlement density (Pearson's r = −0.268, *p* = 0.561, n = 7). Germination success was highest on the fifth (18^th^ Mar) sample period, and interestingly gametophyte size (Figure 4C) also reached a maximum in the same sampling period. Eighteen hrs after release, gametophytes were all a similar size except those collected during the third (19^th^ Feb) sample period which were significantly smaller than those from fifth (18^th^ Mar).

### Gametophyte survival and growth after culture

**Figure 5:**
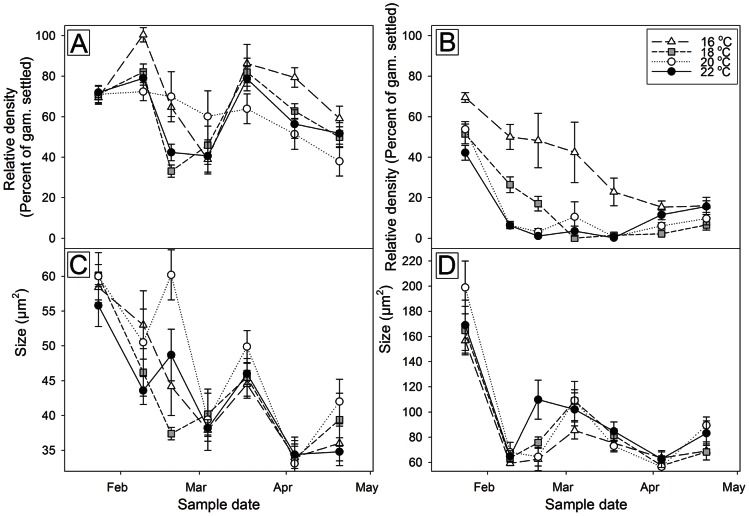
The mean (±SE) relative density and size of gametophytes. Plots represent relative density measured after 3 (A) and 6 (B) days of culture, and the mean (± SE) size of gametophytes measured after 3 (C) and 6 (D) days of culture. Gametophytes were grown in four different temperatures; 16°C (white triangle, long dash line), 18°C (grey square, small dash line), 20°C (white circle, dotted line), 22°C (black circle, solid line).

The densities and growth of gametophytes after 3 and 6 days in culture were significantly affected by when they were collected during the three month sporulation period (Table 1). The survival of gametophytes after 3 days of culture was highest for the samples collected during the second (9^th^ Feb) and fifth (18^th^ Mar) sample periods; however, between these periods there was a decline (Figure 5A). For gametophytes counted after 6 days of culture (Figure 5B), survival was highest at the beginning of the season and then generally declined throughout. There was a significant correlation (Pearson's r = 0.973, *p*<0.001, n = 7) between the gametophyte density after 3 days of culture and initial zoospore settlement density; however, after 6 days this relationship had weakened and was no longer significant (Pearson's r = 0.704, *p* = 0.078, n = 7).

**Table 1:** Analysis of Variance of gametophyte density and size.

		Day 3			Day 6		
Source of variation	df	MS	F	*p*	MS	F	*p*
Density							
Sample Period	6	127.27	342.21	**<0.001**	118.21	236.19	<0.001
Culture Temp.	3	3.74	2.76	0.072	28.24	6.45	0.004
S. Period*Temp	18	1.35	3.64	**<0.001**	4.38	8.75	<0.001
Residual	812	0.37			0.50		
Size							
Sample Period	6	16.50	31.66	**<0.001**	311.25	42.34	<0.001
Culture Temp.	3	2.13	2.21	0.122	30.21	2.65	0.080
S. Period*Temp	18	0.96	1.85	**0.025**	11.39	1.55	0.082
Residual	140	0.52			7.35		

ANOVA testing the relationship between sample period (random factor; seven fortnights) and culture temperature (fixed factor; four categories 16, 18, 20, 22°C) after 3 and 6 days of culture.

The density of gametophytes after 3 days was not significantly affected by culture temperature (Table 1). Densities were generally higher in the 16°C (Figure 5A), and in sample periods (fortnights) where there was a significant difference between temperature treatments, density was always highest in the 16°C treatment (Table 2). After 6 days there was a significant difference between temperatures, and a visual comparison of the results indicated that the highest densities occurred the 16°C treatment compared to all other treatments (Figure 5B). This was supported by the pairwise comparisons which revealed that in all fortnights expect one, and in all fortnights where there was a significant difference in density between temperatures, 16°C always produced the highest survival (Table 2). The temperature treatment which returned the poorest survival was more variable, with 18, 20, and 22°C all returning the lowest densities in at least one fortnight after both 3 and 6 days of culture, and this is likely the reason for the significant interaction term in the ANOVA.

**Table 2:** Summary of pairwise SNK comparisons of temperature treatments (°C) within each sample period.

Sample period	Density		Size	
	Day 3	Day 6	Day 3	Day 6
1	18 = 16 = 20 = 22	22 = 18 = 20 = 16	22 = 16 = 20 = 18	16 = 18 = 22 = 20
2	20 = 22 = 18 = 16	**20 = 22<18<16**	22 = 18 = 20 = 16	**16 = 20 = 18<22**
3	**18 = 22<20 = 16**	**22 = 20<18<16**	**18 = 16 = 22<20**	16 = 22 = 20 = 18
4	22 = 16 = 18 = 20	18 = 22 = 20 = 16	16 = 22 = 20 = 18	20 = 18 = 22 = 16
5	20 = 16 = 18 = 22	**22 = 20 = 18<16**	16 = 18 = 22 = 20	20 = 16 = 22 = 18
6	**20 = 22<18 = 16**	**18<20<22 = 16**	20 = 18 = 16 = 22	18 = 16 = 20 = 22
7	**20<18 = 22 = 16**	18 = 20 = 16 = 22	22 = 16 = 18 = 20	**16<18 = 22 = 20**

Within each sample period (random) the temperature (fixed) treatments are ranked based on their means (lowest to highest), and are highlighted in bold where a significant difference (p<0.05) existed. An ‘ = ’ symbol is used to show where sample means are not significantly different, and where there was a difference between treatments, ‘<’ is used to highlight which temperatures are greater than which other treatments.

During the zoospore release period (January to April), the size of gametophytes after 3 and 6 days of culture was significantly different among dates of collection (Table 1). Gametophyte size was greatest on the very first sampling occasion after both culture periods (Figure 5C and D). There was no significant correlation between initial spore size and gametophyte size after 3 (Pearson's r = 0.438, *p* = 0.326, n = 7) or 6 (Pearson's r = 0.322, *p* = 0.481, n = 7) days of culture.

Culture temperature did not have a significant effect on size of gametophytes after both 3 and 6 days in culture (Table 1). The smallest gametophytes were generally recorded in the 16 and 18°C treatments (Figure 5C and D); and in most samples the best growth was recorded in 20 and 22°C. In all fortnights where there was a significant difference between temperature treatments either 20°C or 22°C always returned the highest growth, while the smallest gametophytes were always recorded in either 16°C or 18°C (Table 2). The variability in the temperature which returned the highest and lowest growth is likely to have caused the lack of significant difference between culture temperatures.

### Environmental conditions

**Figure 6:**
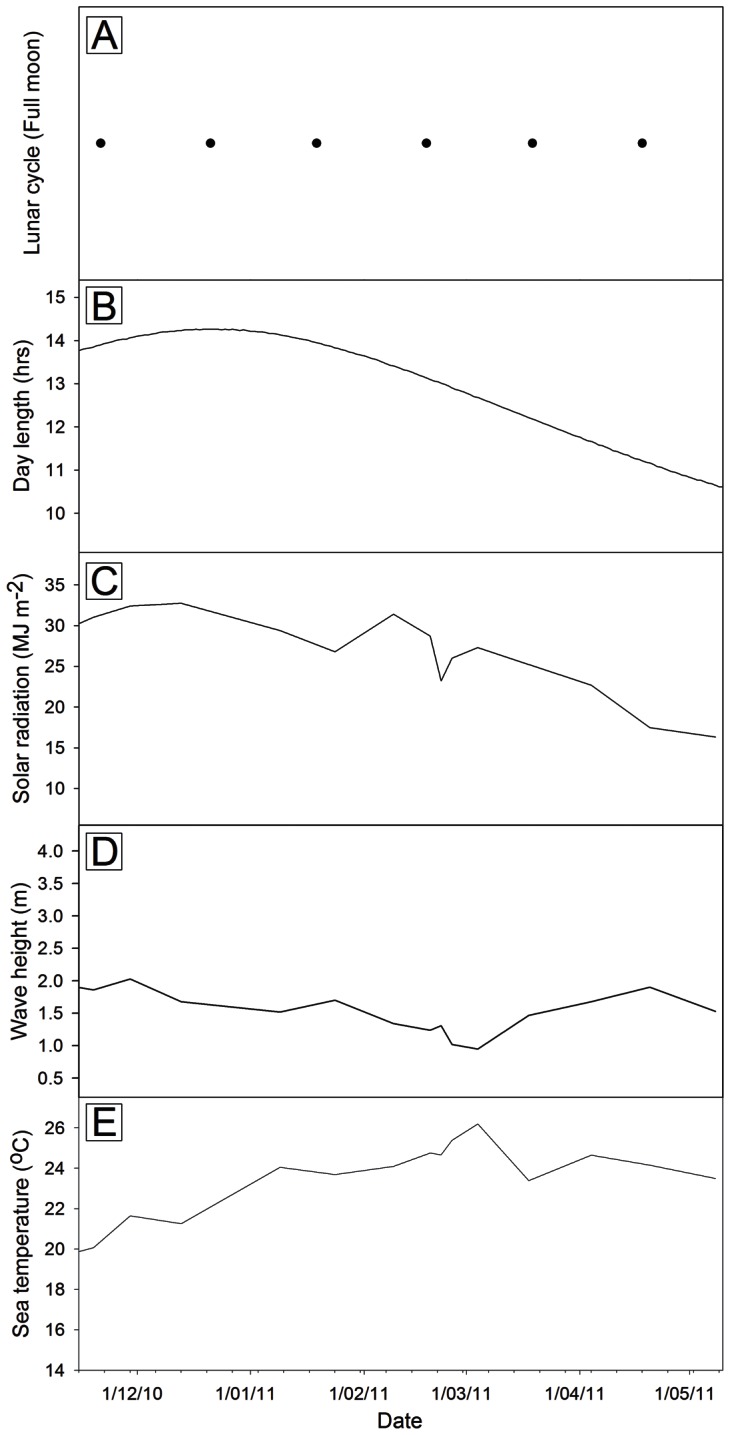
Environmental conditions across the sampling period. A) Full moon cycles (Source: Sutherland Shire Weather Station) B) Day-length (Photoperiod) (Source: Geoscience Australia); C) Global solar radiation (Source: Australian Bureau of Meteorology); D) Wave height (Source: Western Australian Department of Transport); E) Sea temperature (Source: Western Australian Department of Fisheries).

The only significant relationship detected when environmental conditions (Figure 6) were compared to initial settlement data, as well as gametophyte growth and survival was between gametophyte size after 3 days of culture and daylength (Table 3). There were no significant regressions (*p*>0.05) between the environment and initial spore size, density and percent germinated or for density after three and six days in culture. *In situ* sea temperature was not correlated with gametophyte growth or survival although it varied among the sample periods from 21.5 to 26.6°C during the zoospore release period.

**Table 3:** Summary of linear regressions of biological variables against environmental variables.

Environmental variable	r^2^	F	*p*
Initial density			
Sea Temperature	0.270	1.85	0.232
Swell Height	0.236	1.54	0.269
Day Length	0.100	0.55	0.490
Solar Radiation	0.033	0.17	0.695
Initial size			
Sea Temperature	0.102	0.57	0.486
Swell Height	0.068	0.36	0.572
Day Length	0.095	0.53	0.502
Solar Radiation	0.098	0.50	0.495
Percent germinated			
Sea Temperature	0.007	0.03	0.863
Swell Height	0.336	2.54	0.172
Day Length	0.065	0.35	0.581
Solar Radiation	0.305	2.30	0.198
Density Day 3			
Sea Temperature	0.253	1.69	0.250
Swell Height	0.104	0.58	0.481
Day Length	0.185	1.14	0.335
Solar Radiation	0.123	0.70	0.440
Density Day 6			
Sea Temperature	0.189	1.16	0.330
Swell Height	0.104	0.58	0.481
Day Length	0.462	4.29	0.093
Solar Radiation	0.081	0.44	0.536
Size Day 3			
Sea Temperature	0.256	1.72	0.246
Swell Height	0.000	0.00	0.995
**Day Length**	**0.700**	**11.69**	**0.019**
Solar Radiation	0.290	2.05	0.212
Size Day 6			
Sea Temperature	0.016	0.08	0.789
Swell Height	0.010	0.05	0.831
Day Length	0.321	2.36	0.185
Solar Radiation	0.013	0.07	0.805

Regressions of initial size, density, number germinated and density and size after 20 and 6 days against four environmental variables.

Over the study, the full moon occurred on the 18^th^ or 19^th^ of each month (Figure 6). Peaks in zoospore release densities coincided with the full moon phase of that month, and in the middle of the reproductive season, both sites recorded few zoospores and this coincided with the timing of the new moon. Settlement density and survival after 3 days followed similar patterns to zoospore release densities, thus high densities coincided with the full moon phase of that month. Initial gametophyte size and germination success did not reflect lunar patterns at the time of sampling. Growth rates after 3 days of culture also peaked at similar times to the full moon; however, growth after 6 days did not.

## Discussion

This study revealed distinct temporal patterns in the performance of the early life-stages of kelp collected from temperate Western Australia. *Ecklonia radiata* was reproductive between January and April [43] with high densities of zoospores and optimal growth and survival of gametophytes occurring at the beginning of the reproductive season. Gametophyte growth could be related to daylength, suggesting that *E. radiata* released its healthiest zoospores when days were longest for better germination, growth, and sporophyte production. The influence of culture temperature varied somewhat throughout the reproductive season; however, highest survival occurred in low temperatures, while greatest increase in size was recorded in 20 and 22°C. These findings suggest that zoospores are released when conditions promote growth, rather than maximise survival, perhaps giving *E. radiata* a competitive advantage over other species, reducing intra-specific competition for space among developing gametophytes.

Zoospore concentrations were extremely variable over the release period, and this, in turn, resulted in highly varied settlement densities for culture. Initial settlement densities can have both positive and negative effects on gametophyte growth and survival; for example, in higher densities there is increased competition for nutrients and space [49], [50]. In this study, patterns in survival after 3 days of culture were correlated with initial densities; however, this was not the case after 6 days. Reed *et al.* [50] found that when gametophyte densities were high and crowding existed, growth and survival were limited and reflected competition for space and resources. While it is possible that patterns in survival may be a result of crowding, survival after 6 days, as well as growth after both 3 and 6 days, was not significantly correlated with initial densities. It is, therefore, unlikely that the patterns in size and survival observed in this study were a result of gametophyte density.

*Ecklonia radiata* gametophyte growth and survival was not generally significantly linearly related to a range of *in situ* environmental variables (sea temperature, swell height, daylength, solar radiation). Only growth (measured as gametophyte size after 3 days) was related with daylength. In Laminariales, gametophytes are highly sensitive to fluctuations in light intensity [19], [25], [26] and in some cases excessive shading during winter can limit gametophyte growth [51]. The early growth rates of *E. radiata* gametophytes in temperate Western Australia were highest at the beginning of the sporulation period, and this was linked to the longest days. Kelp gametophytes were released when days were longest, so they were exposed to light for longer periods promoting photosynthesis and better growth and survival [52], [53]. Choi *et al.* [53] found that *Undaria pinnatifida* required a long day length for gametophyte growth, and a shorter day length for sporophyte production. Since it takes approximately three weeks for *E. radiata* gametophytes to mature [23], the release of the healthiest gametophytes may occur early, when days are long to promote germination and growth, but reproduction and the production of young sporophytes occurs later when daylengths are shorter and water temperature declining.

Culture temperature had an effect on gametophyte growth and survival, which has also been shown by other authors [27], [54]. In this study, gametophytes cultured in 16°C appeared to have the greatest survival, while the treatments returning poorest survival alternated throughout the season but were generally the 20 and 22°C temperatures. In contrast, gametophytes cultured in the two lower temperatures, 16 and 18°C grew the slowest, while the temperature treatment which promoted optimum growth alternated between 20 and 22°C. During this study sea temperatures ranged from 21.5–26.6°C, thus it appears that gametophytes grow in waters that are well above the thermal optima for survival found here, but these conditions are better for growth. This indicates that zoospores are released during periods when water temperatures would optimise growth rather than maximise survival. This suggests an interesting ecological trade-off that gives priority to growth over survival, perhaps because kelp recruitment is not propagule limited [14] and the increased growth rate gives *E. radiata* a competitive advantage for space over other species, and reduces intraspecific competition driven by high gametophyte densities.

*Ecklonia radiata* reproduction was occurring when temperatures were extreme, and gametophytes grew well in high temperatures. Unlike most Laminariales, this species is able to tolerate high temperatures [28]. Only a few species (*Eisenia bicyclis, Laminaria japonica, Costaria costata and Undaria pinnatifida*), all collected from Japan, have been shown to survive beyond the temperatures which *E. radiata* endured *in situ* during this study [28]. Without further experimentation, the upper thermal limits of *E. radiata* remain unknown, but it seems likely that *E. radiata* may be able to survive in some of the highest temperature regimes for Laminariales. Clearly, *E. radiata* gametophytes are well adapted to live within the thermal extremes of its distribution in Australia. It is plausible that the response to temperature that *E. radiata* gametophytes exhibited here is related to biogeography, since this species spans a wide latitudinal range in Australia (26°S–43°S) and samples were collected towards the centre of its distribution. At the southern (cool-water) limits of its distribution, *E. radiata* reproduces in waters that rarely exceed 16°C, while in northern populations, reproduction is occurring in waters in excess of 22°C. Thus, *E. radiata* may exhibit general thermal tolerance levels that allow all populations to achieve optimum performance irrespective of biogeography. Studies investigating the thermal tolerances of Laminariales have most commonly reported a lack in variability across species' distribution [54], [55]. This raises some poignant questions regarding the thermo-tolerance characteristics of geographically separated populations of *E. radiata* in Australia, and whether the results found here can be extrapolated to other *E. radiata* populations in different regions across the continent. It is possible that thermo-tolerance varies across the distribution of this species, but it is also just as likely that thermally adapted populations will exhibit similar patterns of seasonality and change over time.

The effect of culture temperature did vary over the reproductive season, with a significant interaction between culture temperature and collection time for both gametophyte growth and survival. Over the whole reproductive season gametophyte survival was greatest in 16°C and growth was optimal in either 20 or 22°C. Despite water temperatures exceeding 26°C during *E. radiata* reproduction in southwest Australia, survival was optimal at 10°C lower than ambient temperatures. Tala *et al.* [29] speculated that the intrinsic characteristics of *Lessonia trabeculata* gametophytes are defined by the surrounding environment, and that juveniles would exhibit tolerances based on the conditions adult populations were subjected to. This does not appear to apply to *E. radiata*; if this was the case, gametophytes would exhibit some physiological differences depending on the timing of production respective to the surrounding climate.

Temperature and light are well-documented drivers of Laminariales reproduction, and both had an effect on the early survival and growth of *E. radiata*. Early growth rates of gametophytes were positively related to day length, with the fastest growing zoospores released when the days were longest so that gametophytes received the maximum amount of light for growth. Survival was greatest in the coolest temperature, while optimum growth was achieved in higher temperatures. Since reproduction occurred when *in situ* sea temperatures were highest, an ecological trade-off may be occurring whereby growth is prioritised over survival. The relationship between culture temperature and gametophyte size and density remained constant irrespective of ambient sea temperatures at the time of collection. Since there was little-to-no obvious *in situ* temperature signal in the culture of *E. radiata*, this suggests that gametophytes of this species are thermally tolerant to high and variable temperatures.

## References

[1] Steneck RS, Graham MH, Bourque BJ, Corbett D, Erlandson JM, et al (2002) Kelp forest ecosystems: biodiversity, stability, resilience and future. Environmental Conservation 29: 436–459.

[2] Schiel DR, Foster GG (1986) The structure of subtidal algal stands in temperate waters. Oceanography and Marine Biology: Annual Review 24: 265–307.

[3] Wernberg T, Kendrick GA, Toohey BD (2005) Modification of the physical environment by an *Ecklonia radiata* (Laminariales) canopy and implications for associated foliose algae. Aquatic Ecology 39: 419–430.

[4] Graham MH (2004) Effects of local deforestation on the diversity and structure of Southern California giant kelp forest food webs. Ecosystems 7: 341–357.

[5] Vanderklift MA, Lavery PS, Waddington KI (2009) Intensity of herbivory on kelp by fish and sea urchins differs between inshore and offshore reefs. Marine Ecology Progress Series 376: 203–211.

[6] Schiel DR, Foster MS (2006) The population biology of large brown seaweeds: ecological consequences of multiphase life histories in dynamic coastal environments. Annual Review of Ecology, Evolution and Systematics 37: 343–372.

[7] Coelho SM, Rijstenbil JW, Brown MT (2000) Impacts of anthropogenic stresses on the early development stages of seaweeds. Journal of Aquatic Ecosystem Stress and Recovery 7: 317–333.

[8] Harley CDG, Hughes RA, Hultgren KM, Miner BG, Sorte C, J B, et al (2006) The impacts of climate change in coastal marine systems. Ecology Letters 9: 228–241. 16958887 10.1111/j.1461-0248.2005.00871.x

[9] Wernberg T, Russell BD, Moore PJ, Ling SD, Smale DA, et al (2011) Impacts of climate change in a global hotspot for temperate marine biodiversity and ocean warming. Journal of Experimental Marine Biology and Ecology 400: 7–16.

[10] Larkum AWD (1986) A study of growth and primary production in *Ecklonia radiata* (C. Ag.) J. Agardh (Laminariales) at a sheltered site in Port Jackson, New South Wales. Journal of Experimental Marine Biology and Ecology 96: 177–190.

[11] Russell BD, Harley CDG, Wernberg T, Mieszkowska N, Wddicombe S, et al (2012) Predicting ecosystem shifts requires new approaches that integrate the effects of climate change across entire systems. Biology Letters 8: 164–166. 21900317 10.1098/rsbl.2011.0779 PMC3297386

[12] Fritsch FD (1942) Studies in the comparitive morphology of the algae II: The algal life cycle. Annals of Botany 24: 533–563.

[13] McConnico LA, Foster MS (2005) Population biology of the intertidal kelp, *Alaria marginata* Postels and Ruprecht: A non-fugitive annual. Journal of Experimental Marine Biology and Ecology 324: 61–75.

[14] Joska MAP, Bolton JJ (1987) *In situ* measurement of zoospore release and seasonality of reproduction in *Ecklonia maxima* (Alariaceae, Laminariales). European Journal of Phycology 22: 209–214.

[15] Reed DC, Ebeling AW, Anderson TW, Anghera M (1996) Differential reproductive responses to fluctuating resources in two seaweeds with different reproductive strategies. Ecology 77: 300–316.

[16] Buschmann AH, Moreno C, Vasquez JA, Hernandez-Gonzalez MC (2006) Reproduction strategies of *Macrocystis pyrifera* (Phaeophyta) in Southern Chile: The importance of population dynamics. Journal of Applied Phycology 18: 575–582.

[17] Buchholz CM, Lüning K (1999) Isolated, distal blade discs of the brown alga *Laminaria digitata* form sorus, but not discs, near to the meristematic transition zone. Journal of Applied Phycology 16: 579–584.

[18] Reed DC (1987) Factors affecting the production of sporophylls in the giant kelp *Macrocystis pyrifera* (L.) C.Ag. Journal of Experimental Marine Biology and Ecology 113: 61–69.

[19] Thornber CS, Kinlan BP, Graham MH, Stachowicz JJ (2004) Population ecology of the invasive kelp *Undaria pinnatifida* in California: environmental and biological controls on demography. Marine Ecology Progress Series 268: 69–80.

[20] Reed DC, Anderson TW, Ebeling AW, Anghera M (1997) The role of reproductive synchrony in the colonization potential of kelp. Ecology 78: 2443–2457.

[21] Amsler CD, Neushul M (1989) Diel periodicity of spore release from the kelp *Nereocystis luetkeana* (Mertens) Postels *et* Ruprecht. Journal of Experimental Marine Biology and Ecology 134: 117–127.

[22] Reed DC, Brzezinski MA, Coury DA, Graham WM, Petty RL (1999) Neutral lipids in macroalgal spores and their role in swimming. Marine Biology 133: 737–744.

[23] Fritsch FE (1952) Structure and reproduction of algae. London: Cambridge University Press.

[24] Fredersdorf J, Müller R, Becker S, Wiencke C, Bischof K (2009) Interactive effects of radiation, temperature and salinity on different life history stages of the Arctic kelp *Alaria esculenta* (Phaeophyceae). Oecologia 160: 483–492. 19330357 10.1007/s00442-009-1326-9

[25] Bolton JJ, Levitt GJ (1985) Light and temperature requirements for growth and reproduction in gametophytes of *Ecklonia maxima* (Alariaceae: Laminariales). Marine Biology 87: 131–135.

[26] Lüning K (1980) Critical levels of light and temperature regulating the gametogenesis of three *Laminaria* species (Phaeophyceae). Journal of Phycology 16: 1–15.

[27] Novaczek I (1984) Response of gametophytes of *Ecklonia radiata* (Laminariales) to temperature in saturating light. Marine Biology 82: 241–245.

[28] Dieck IT (1993) Temperature tolerance and survival in darkness of kelp gametophytes (Laminariales, Phaeophyta): ecological and biogeographical implications. Marine Ecology Progress Series 100: 253–264.

[29] Tala F, Edding M, Vasquez JA (2004) Aspects of the reproductive phenology of *Lessonia trabeculata* (Laminariales: Phaeophyceae) from three populations in northern Chile. New Zealand Journal of Marine and Freshwater Research 38: 255–266.

[30] Lee JA, Brinkuis BH (1988) Seasonal light and temperature interaction effects on development of *Laminaria saccharina* (Phaeophyta) gametophytes and juvenile sporophytes. Journal of Phycology 24: 181–191.

[31] Wernberg T, Vanderklift MA, How J, Lavery PS (2006) Export of detached macroalgae from reefs to adjacent seagrass beds. Oecologia 147: 692–701. 16323014 10.1007/s00442-005-0318-7

[32] Steinberg PD, Kendrick GA (1999) Kelp Forests. In: Andrew NL, editor. Under Southern Seas. Australia: University of New South Wales. pp. 60–71.

[33] Kennelly SJ, Larkum AWD (1983) A preliminary study of temporal variation in the colonization of subtidal algae in an *Ecklonia radiata* community. Aquatic Botany 17: 275–282.

[34] Crawley KR, Hyndes GA (2007) The role of different types of detached macrophytes in the food and habitat choice of a surf-zone inhabiting amphipod. Marine Biology 151: 1433–1443.

[35] Fletcher WJ (1987) Interactions among subtidal Australian sea urchins, gastropods, and algae: effects of experimental removals. Ecological Monographs 57: 89–109.

[36] Tuya F, Wernberg T, Thomsen MS (2008) The spatial arrangement of reefs alters the ecological patterns of fauna between interspersed algal habitats. Estuarine, Coastal, and Shelf Science 78: 774–782.

[37] Smith SDA, Simpson RD, Cairns SC (1996) The macrofaunal community of *Ecklonia radiata* holdfasts: Description of the faunal assemblage and variation associated with differences in holdfast volume. Australian Journal of Ecology 21: 81–95.

[38] Smale DA, Kendrick GA, Wernberg T (2010) Assemblage turnover and taxonomic sufficiency of subtidal macroalgae at multiple spatial scales. Journal of Experimental Marine Biology and Ecology 384: 76–86.

[39] Wernberg T, Kendrick GA, Phillips JC (2003) Regional differences in kelp-associated algal assemblages found on temperate limestone reefs in southwestern Australia. Diversity and Distributions 9: 427–441.

[40] Bidwell JR, Wheeler KW, Burridge TR (1998) Toxicant effects on the zoospore stage of the marine macroalga *Ecklonia radiata* (Phaeophyta: Laminariales). Marine Ecology Progress Series 163: 259–265.

[41] Jennings R (1967) The development of the gametophyte and young sporophyte of *Ecklonia radiata* (C.Ag.) J.Ag. (Laminariales). Journal of the Royal Society of Western Australia 50: 93–96.

[42] Smale DA, Wernberg T (2009) Satellite-derived SST data as a proxy for water temperature in nearshore benthic ecology. Marine Ecology Progress Series 387: 27–37.

[43] Mohring MB, Wernberg T, Kendrick GA, Rule MJ (2013) Reproductive synchrony in a habitat-forming kelp and its relationship with environmental conditions. Marine Biology 160: 119–126.

[44] Provasoli L (1968) Media and prospects for the cultivation of marine algae. In: Watanabe A, Hattori A, editors. Cultures and Collections of Algae. Hakone, Japan: Japanese Society Plant Physiolology. pp. 63–75.

[45] Rasband W (2009) ImageJ 1.42q. U.S.A.: National Institutes of Health.

[46] Underwood AJ (1997) Experiments in ecology: their logical design and interpretation using analysis of variance. Cambridge: Cambridge University Press. 504 p.

[47] Underwood AJ, Chapman MG (1998) GMAV5 for WINDOWS. Australia: Institute of Marine Ecology, University of Sydney.

[48] Minitab Inc. (2000) MINITAB 13.1. Pennsylvania: State University.

[49] Steen H (2003) Intraspecific competition in *Sargassum muticum* (Phaeophyceae) germlings under various density, nutrient and temperature regimes. Botanica Marina 46: 36–43.

[50] Reed DC, Neushul M, Ebeling AW (1991) Role of settlement density on gametophyte growth and reproduction in the kelps *Pterygophora californica* and *Macrocystis pyrifera* (Phaeopyceae). Journal of Phycology 27: 361–366.

[51] Kirkman H (1981) The first year in the life history and the survival of the juvenile marine macrophyte, *Ecklonia radiata* . Journal of Experimental Marine Biology and Ecology 55: 243–254.

[52] Nelson WA (2005) Life history and growth in culture of the endemic New Zealand kelp *Lessonia variegata* J. Agardh in response to differing regimes of temperature, photoperiod and light. Journal of Applied Phycology 17: 23–28.

[53] Choi HG, Kim YS, Lee SJ, Park EJ, Nam KW (2005) Effects of daylength, irradiance and settlement density on the growth and reproduction of *Undaria pinnatifida* gametophytes. Journal of Applied Phycology 17: 423–430.

[54] Bolton JJ, Lüning K (1982) Optimal growth and maximal survival temperatures of Atlantic *Laminaria* species (Phaeophyta) in culture. Marine Biology 66: 89–94.

[55] Ladah LB, Zertuche-González JA (2007) Survival of microscopic stages of a perennial kelp (*Macrocystis pyrifera*) from the center and the southern extreme of its range in the Northern Hemisphere after exposure to simulated El Niño stress. Marine Biology 152: 677–686.

